# Development and preliminary evaluation of a tele-rehabilitation exercise system using computer-generated animation

**DOI:** 10.20407/fmj.2021-020

**Published:** 2022-01-25

**Authors:** Nobuhiro Kumazawa, Soichiro Koyama, Masahiko Mukaino, Kazuhiro Tsuchiyama, Tsuyoshi Tatemoto, Hiroki Tanikawa, Kei Ohtsuka, Masaki Katoh, Yohei Otaka, Eiichi Saitoh, Shigeo Tanabe

**Affiliations:** 1 Graduate School of Health Sciences, Fujita Health University, Toyoake, Aichi, Japan; 2 Faculty of Rehabilitation, School of Health Sciences, Fujita Health University, Toyoake, Aichi, Japan; 3 Department of Rehabilitation Medicine I, School of Medicine, Fujita Health University, Toyoake, Aichi, Japan; 4 Tokyo Bay Rehabilitation Hospital, Narashino, Chiba, Japan; 5 Department of Rehabilitation, Fujita Health University Hospital, Toyoake, Aichi, Japan

**Keywords:** Tele-rehabilitation, Exercise, Computer-generated animation

## Abstract

**Objectives:**

To evaluate the safety and acceptability of a newly developed tele-rehabilitation exercise system using computer-generated animation.

**Methods:**

The participants comprised a convenience sample of 38 diverse individuals in Experiment 1 (15 healthy young people, 16 healthy older people, 5 patients with stroke, and 2 patients with respiratory disease) and 18 healthy older individuals in Experiment 2. Experiment 1 assessed safety in terms of cardiopulmonary vascular aspects and risk of fall, and Experiment 2 assessed treatment acceptability via a subjective evaluation. All participants completed the same exercise program. The safety assessment was conducted using heart rate (HR) and saturation of percutaneous oxygen (SpO2), measured before and after exercise. In addition, the occurrence of falls was assessed. For the acceptability assessment, the participants answered five questions (three-point Likert scale) after the exercise program.

**Results:**

The safety assessment indicated that HR and SpO2 changed from 70.5±10.2 beats per minute and 97.8±1.3% before exercise to 87.6±13.6 beats per minute and 98.2±0.9% after exercise, respectively. In addition, all participants completed the exercises without experiencing any falls. In the acceptability assessment, the score reflecting continuation desire was the highest of the five items examined (2.71±0.46). In contrast, the adequacy of exercise intensity had the lowest score (1.29±0.57).

**Conclusions:**

The present system was confirmed to be safe, and the participants were motivated to continue the exercises. Future developments should incorporate a function to enable participants and medical staff to adjust exercise intensity according to individual physical function.

## Introduction

Demographic aging is occurring in developed societies because of a decrease in birth and mortality rates and an increase in life expectancy.^1^ In 2015, older individuals (>65 years of age) made up 26.6% of the population in Japan,^2^ and this proportion is expected to increase to approximately 33.3% in 2036.^2^ Globally, 727 million people in 2020 were estimated to be aged 65 years and older, and this is projected to more than double to over 1.5 billion by 2050. The proportion of the world’s older population is expected to increase continuously from 9.3% in 2020 to 16.0% in 2050. By mid-century, one in six people worldwide will be aged 65 years or older.^3^

The activities of daily living (ADL) are commonly limited in older people due to a decline in various aspects of physical function, such as muscle weakness,^4,5^ reduced joint flexibility,^6^ and decreased motor endurance.^7,8^ To enable older individuals to continue living at home in the community, various strategies for providing preventive and therapeutic support to maintain physical function are necessary.

A previous meta-analysis showed that exercise therapy for older people improved physical function.^9^ As a means of exercise therapy, tele-rehabilitation has recently attracted the attention of researchers. Tele-rehabilitation enables people to remotely access rehabilitation services in their homes using real-time telecommunication technology, and had been found to prevent the decline of physical function and the ADL.^10^ Accordingly, it has been applied with the aim of reducing the length of hospital stays and lowering economic costs for patients and healthcare providers, and also to provide easy access to rehabilitation facilities from patients’ homes.^10^ Various tele-rehabilitation systems specialized for individual disorders have been proposed. For instance, previous studies have indicated that tele-rehabilitation systems had positive effects in patients with physical and psychological health conditions, such as knee replacement,^11,12^ breast cancer surgery,^13^ stroke,^14^ speech disorders,^15–17^ mental health issues,^18^ wounds,^19–21^ and neurological diseases.^22^

However, a tele-rehabilitation system for use by a wider range of older people has not been developed. Older individuals commonly have various comorbid disorders and large variability in physical function and endurance. Especially, many older people show a decline in cardiopulmonary function and balance ability that increases their risk of falls.^23–25^ Previous studies have suggested that habitual physical activity can improve cardiopulmonary function and balance ability.^26,27^ Thus, to develop a suitable tele-rehabilitation system for older individuals, exercise intensity and content should be optimized from the perspective of adequate safety, particularly in terms of cardiopulmonary load and fall risk.

In addition, the program contents must be easily understood by the users to ensure appropriate implementation of tele-rehabilitation. Because older people commonly have reduced audiovisual abilities,^28–30^ the information contained in the tele-rehabilitation program must be easy to discern and interpret. As a means of communication, computer-generated (CG) animation is expected to be useful in the application of tele-rehabilitation. Using CG animation, it is easy to edit features of graphics, such as enlarging a character’s body parts and/or adding arrows to encourage the user to pay extra attention to certain elements, thus improving their understanding of the exercises.

In the present study, we developed a tele-rehabilitation exercise system using CG animation and assessed it in terms of feasibility from the perspective of safety and acceptability including ease of interpretation.

## Methods

### Participants and study design

We conducted two experiments, one to confirm safety (Experiment 1) and one to assess the acceptability (Experiment 2) of the exercise program. A convenience sample of 38 people participated in Experiment 1 (mean age 53.7±25.9 years, 21 males, 17 females): 15 healthy young people (mean age 23.9±2.5 years, 7 males, 8 females), 16 healthy older people (mean age 76.2±5.3 years, 10 males, 6 females), 5 patients with stroke (mean age 63.0±20.3 years, 2 males, 3 females), and 2 patients with respiratory disease (mean age 75.0±5.0 years, 2 males). The patient characteristics are shown in Table 1. A convenience sample of 18 healthy older people participated in Experiment 2 (mean age 64.4±19.5 years, 5 males, 13 females). All participants in both experiments had not taken β-blockers. All participants were informed of the procedures prior to participation and provided written and verbal informed consent. The present study was approved by our University Research Ethics Committee in accordance with the Declaration of Helsinki (HM17-312, HM18-027, HM18-310).

### Characteristics of the proposed tele-rehabilitation exercise system

**Figure 1:**
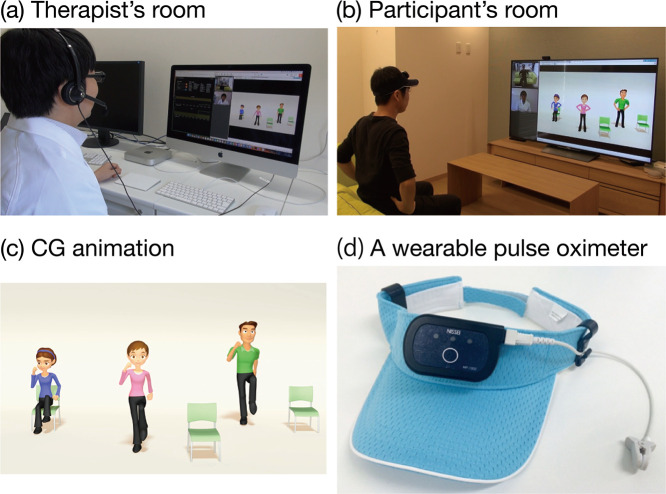
Proposed tele-rehabilitation exercise system. The proposed system was a television-type communication device. Using a desktop microphone and speakers, the users could communicate remotely with medical staff (a and b). The system provided an effective and enjoyable exercise experience using CG animation (c). Real-time physiological condition (HR and SpO2) was monitored using a wearable device (d). Medical staff used these measurements to check exercise tolerance.

We used a newly developed tele-rehabilitation exercise system in the present study (Figure 1). The basic concept of this system was to connect people in their own home with medical staff at a medical institution via the internet and to provide exercise guidance via CG animation. The system enables users to perform exercises according to the movements of CG-animated characters displayed on a monitor, along with recorded therapists’ explanations regarding movements. The system includes new functions, such as enlargements of the character’s body parts and the addition of arrows, as well as the simultaneous display of the character in multiple positions (sitting and standing). Each user’s movements were video recorded and transmitted to the supervising medical staff in real-time for safety monitoring and to provide appropriate movement guidance. For enhanced safety, a wearable pulse oximeter was used during exercise. Each user’s heart rate (HR) and saturation of percutaneous oxygen (SpO2) were displayed on the screen for the benefit of the supervising medical staff. The medical staff members were able to talk to the user and provide guidance regarding the most safe and appropriate movements according to the user’s HR and SpO2. Using these functions, the present system enables medical staff to facilitate safe and acceptable exercises for older or disabled people with a wide variety of physical abilities.

### Experimental setup

A video meeting system (OmniJoin; Brother Industries, Ltd., Aichi, Japan) was used for CG animation video-sharing and communication between the users and medical staff. Prior to each experiment, to reduce the burden on users, the system was installed on a PC and connected to a desktop microphone, speakers, and large monitor. A wearable pulse oximeter (MP-1000; Nihon Seimitsu Sokki Co., Ltd., Gunma, Japan) was used to record HR and SpO2. The medical staff used a headset to communicate with the users. The system started automatically after the user pressed the start button, which enabled the user to talk with the medical staff and initiated the recordings of HR and SpO2. When the medical staff started the exercise program on the PC at the medical facility, the CG animation was displayed on the user’s monitor.

### Exercise program

**Figure 2:**
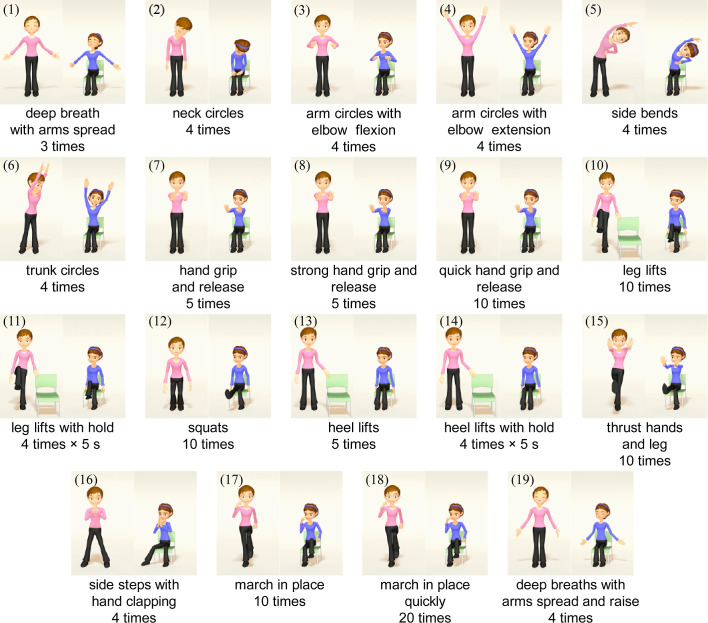
Exercise program: 19 different movements and the number of repetitions. The two standing characters perform the same movements.

The exercise program was developed by certified physical therapists and medical doctors specializing in rehabilitation medicine. The basic concept of the exercise program was to facilitate whole body movements that a wide range of older people, including patients with mild respiratory disease, could perform safely. First, a large number of commonly-used exercises were collected and assessed for suitability from the perspective of physical load and risk of falls. The following specific points were also considered: exercise sequences that started and ended with warm-up and warm-down exercises; exercises that included a wide range of activities such as upper/lower limb and trunk movements, balance exercises, exercises requiring light cognitive load, and continuous movements. As a result, the exercise program in the proposed system consisted of the following 19 different movements (Figure 2): 1) deep breath with arms spread, 2) neck circles, 3) arm circles with elbow flexion, 4) arm circles with elbow extension, 5) side bends, 6) trunk circles, 7) hand grip and release, 8) strong hand grip and release, 9) quick hand grip and release, 10) leg lifts, 11) leg lifts with hold, 12) squats, 13) heel lifts, 14) heel lifts with hold, 15) thrust hands and leg, 16) side steps with hand clapping, 17) march in place, 18) march in place quickly, and 19) deep breaths with arms spread and raised. The total duration of the exercise program was about 12 minutes. Discontinuation of the exercise was determined individually based on subjective symptoms and visual inspection.

### Experiment 1: examination of safety

Before the experiment, the HR at rest was measured to confirm the safety of all participants. All participants were asked to perform the tele-rehabilitation exercise program while in a standing position 2 m from a 55-inch monitor and while watching the CG animation of each exercise. On a computer screen at a remote location, the medical staff observed the participant’s movements, occurrence of falls, HR, and SpO2 during the exercises. Based on this information, the medical staff gave advice to the users to ensure their safe and appropriate movements. The maximum number of participants who could complete the program simultaneously was set to five. In addition, to ensure safety, at least three medical staff members supervised the participants in person at the experimental site. As a measure of safety, HR and SpO2 were recorded before and after the exercises. Some participants who could not maintain a stable standing position were allowed to grasp a chair during the exercises. In addition, a short rest break and a decrease in the number of repetitions were allowed according to each participant’s subjective judgment.

### Experiment 2: examination of acceptability

The experimental environment was set up according to the assumption that the older individuals living in a community used a shared TV. As with Experiment 1, before the experiment began, HR at rest was measured to confirm the safety of all participants. Each participant was asked to perform the tele-rehabilitation exercises in a sitting position on a couch located 1.5 m from a 65-inch monitor, while watching the CG animations of the exercises. During the exercises, the medical staff observed the participant’s movements, HR, and SpO2. To assess acceptability, we designed five questions regarding the user experience of the program, scored using a three-point Likert rating scale (1–3) (Table 2). All participants were asked to answer the questions after performing the tele-rehabilitation exercises.

### Data analysis

For HR and SpO2 in Experiment 1, we calculated the mean values with standard deviations. We used the Wilcoxon signed rank test to compare the values before vs. after the exercises. For the questions in Experiment 2, we calculated the mean values with standard deviations for each item. The scores of the questions were compared using Friedman’s test followed by Bonferroni’s test for multiple comparisons. All statistical analyses were performed using R (version 4.0.5; Institute of Statistical Mathematics, Tokyo, Japan). The statistical significance was set at a p-value of 0.05.

## Results

**Figure 3:**
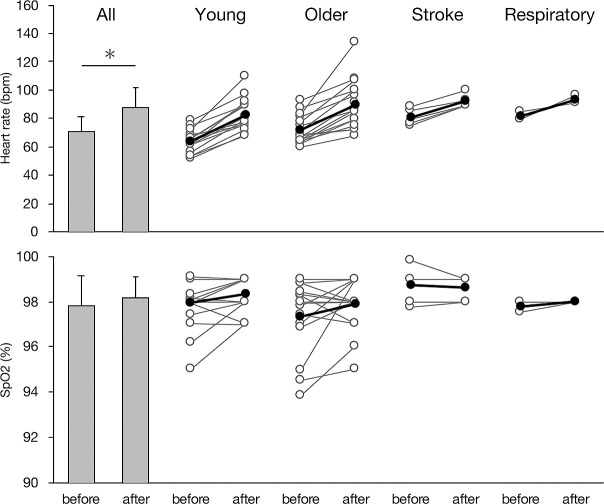
Changes in HR and SpO2. The bars indicate the average HR/SpO2 values before and after the exercises in all participants. Error bars indicate standard deviation. * p<0.05. Gray lines and open circles represent data from individual subjects; black lines and filled circles represent mean data.

**Figure 4:**
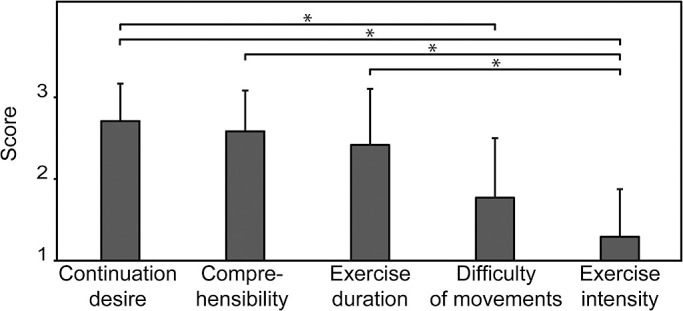
The scores for the questions regarding the user experience of the exercise system. The bars indicate the average values for each question. Error bar indicates standard deviation. * p<0.05.

In all participants, the resting HR was below 120 beats per minute. In both experiments, the participants completed all of the tele-rehabilitation exercises safely, without adverse events including accidents related to falls. The HR and SpO2 values in Experiment 1 are shown in Figure 3. The participant HR was higher after the exercises (87.6±13.6 beats per minute) vs. before the exercises (70.5±10.2 beats per minute) (p<0.05). There was no significant difference in SpO2 before (97.8±1.3%) vs. after the exercises (98.2±0.9%). The scores for the questions in Experiment 2 are shown in Figure 4. The items measuring continuation desire and comprehensibility of the proposed system had high scores (2.71±0.46 and 2.59±0.49, respectively). However, the adequacy of exercise intensity had the lowest score (1.29±0.57). The score for exercise intensity was “suitable” for 2 participants, “low” for 3 participants, and “very low” for 13 participants. The score for difficulty of movements was “suitable” for 4 participants, “easy” for 7 participants, and “very easy” for 7 participants. The score for exercise duration was “suitable” for 9 participants, “short” for 7 participants, and “very short” for 2 participants.

## Discussion

The purpose of this preliminary study was to evaluate the safety and acceptability of the proposed tele-rehabilitation exercise system using CG animation. Our data confirmed that the system was safe for all participants (Experiment 1). Regarding acceptability (Experiment 2), the participants provided relatively positive responses regarding the continuation desire and comprehensibility of the system. Conversely, the exercise intensity was not determined to be sufficient for the older participants in the present study.

From the viewpoint of safety, the proposed tele-rehabilitation exercise program using CG animation was completed by all participants without any adverse events. These results suggest that this system can be used safely from the perspective of cardiopulmonary vascular load and risk of falls. In previous studies, HR and SpO2 have frequently been used as indicators of safety.^31–34^ In the present study, HR was significantly increased after the exercises, but the degree of increase was considered to be within a safe range in all participants according to assessments based on subjective symptoms and visual inspection by the attending medical staff. Conversely, there was no significant difference in SpO2 before vs. after the exercises. In healthy individuals, SpO2 is usually maintained at a constant value of more than 96% during exercise. However, in patients with respiratory or cardiovascular diseases, SpO2 is often decreased during exercise and/or daily activity. Generally, an exercise-induced decrease in SpO2 has a longer time delay than the sensation of dyspnea. That we allowed the participants to rest according to subjective fatigue may have resulted in the lack of change in SpO2 before and after the exercises. In addition, there were no cases of falls or other adverse events. These results suggest that our tele-rehabilitation system can be safely implemented in older people and patients without increasing the risk of falling. Particularly, this program may be suitable for older people and patients with balance disorder, as they are prone to falling in the standing position or walking.^24,25^

Regarding acceptability, relative perspectives related to the items such as advantages or points to be improved were clarified by comparing the related items. The responses indicated that the participants could easily understand the exercises within the proposed system. Our efforts to make the exercises more clearly comprehensible using CG animation might have contributed to the results. In particular, the presentation of magnified images of limbs and arrows to show the relevant body parts and directions of movement might have been influential. Ali et al. discussed the importance of customizable image size for computer animations designed for use by older people, as well as the use of appropriate graphical objects such as icons for attracting attention in people with cognitive problems.^35^ The high comprehensibility of the activities might have led users to give high scores regarding their desire to continue using the tele-rehabilitation system. Conversely, the participants did not have a positive view of the adequacy of exercise intensity. This suggests that it will be necessary to include an additional function to enable the exercise intensity to be adjusted based on individual physical function in future versions of the proposed system. In addition, as some participants answered that the exercise movements were too easy to perform, future exercise programs might benefit from including more complex movements.

The present study indicates that the safety and acceptability of the proposed system using CG animation is sufficient for use in healthy young people, healthy older people, and patients with stroke or respiratory disease. The general purpose of tele-rehabilitation is to improve the accessibility and continuity of rehabilitation for people in geographically remote locations and patients with various disorders.^36^ Tele-rehabilitation also has the potential to save time and reduce economic and human resource costs for health care professionals and facilities.^37^ Thus, the proposed system might broaden the availability of tele-rehabilitation exercises.

The present study has some limitations that merit consideration. First, the installation of the proposed tele-rehabilitation exercise system might be difficult for older people. In the present study, prior to each experiment, the system was installed by a member of the research team. Information and communication technology literacy tends to be lower in older people.^38,39^ Therefore, the development of a simple installation procedure might be needed to promote the use of this system.

Second, regarding safety, the exercise load was only measured before and after the sequence of exercises. However, the maximum HR and minimum SpO2 during exercise might provide more information regarding the exercise intensity. In addition, this study did not assess how much rest was needed for each participant. The frequency and duration of rest during the exercise program should be assessed for future studies of optimal exercise intensity. Furthermore, as a safety function, it might be useful to develop a warning function when a user exceeds the safe limits of the exercise load. From the perspective of the risk of falling, future studies could examine participant balance ability to make the proposed system safer for people with various physical conditions.

Third, regarding acceptability, the present study did not include a control group to examine the effect of CG animation on the comprehensibility of the movements in the tele-rehabilitation exercise program. Further studies should compare videos with human actors and those with CG animation to clarify the effect of the animation. In Experiment 2, the participants were recruited from a convenience sample. Accordingly, only healthy older people participated in the study as the main user group. To examine the generality of user acceptability, future studies should recruit participants with various physical and cognitive conditions with a wide age range.

## Figures and Tables

**Table1:** Patient characteristics (N=7)

Gender	Age (year)	Weight (kg)	Height (m)	BMI (kg/m^2^)	Disease	Severity	Gait independence
M	77	70.9	166.6	25.7	Cerebral Infarction	SIAS-motor function: 4-4-4-4-4	Independence
F	76	57.6	153.0	24.6	Cerebral Hemorrhage	SIAS-motor function: 5-5-5-5-4	Independence
M	72	53.1	165.0	19.5	Cerebral Infarction	SIAS-motor function: 4-3-4-3-3	Independence
F	67	45.1	150.0	20.0	Cerebral Hemorrhage	SIAS-motor function: 4-4-4-4-4	Independence
F	23	55.0	160.0	21.5	Cerebral Hemorrhage	SIAS-motor function: 3-1C-4-4-4	Independence
M	80	51.9	163.5	19.5	Lung Cancer	TNM classification: Stage II	Independence
M	70	51.7	163.0	19.5	Lung Cancer	TNM classification: Stage IV	Independence

BMI, Body Mass Index; M, male; F, female; SIAS, Stroke Impairment Assessment Set; TNM, Tumor-Node-Metastasis

**Table2:** Survey questions regarding the user experience of the exercise system

Question	Answer		
	1	2	3
Exercise intensity	Very high or low	High or low	Suitable
Difficulty of movements	Very difficult or easy	Difficult or easy	Suitable
Comprehensibility	Incomprehensible	Slightly incomprehensible	Easy to understand
Exercise duration	Very long or short	Long or short	Suitable
Continuation desire	No desire to continue	Intermediate	Desire to continue
